# Current status and trend of clinical development of orphan drugs in China

**DOI:** 10.1186/s13023-022-02440-4

**Published:** 2022-07-27

**Authors:** Ziling Xiang, Wengao Jiang, Bo Yan, Junhao Jiang, Hang Zheng

**Affiliations:** School of Pharmacy, Chongqing Medical University, Yuanjiagang Campus, Shiyou Road Street, Yuzhong District, Chongqing, 68485161 China

**Keywords:** Clinical trial, Rare disease, Orphan drugs, ChinaDrugTrials.org, NRDRS

## Abstract

**Background:**

Rare diseases have been increasingly recognized as unmet medical and health needs worldwide; a growing demand for the development of orphan drugs emerges subsequently. Therefore, it is of great interest for both the Chinese regulatory agency and pharmaceutical companies to keep tract on the clinical development of orphan drugs in China.

**Objective and method:**

This study aims to reveal the current situation and trend of the clinical development of orphan drugs in China, based on the data collected from the Chinese official platform, dating from January 1, 2013 to December 31, 2021.

**Results:**

A total of 331 clinical trials for orphan drugs were extracted from the platform, covering 31 rare diseases and 124 drugs. Increases were seen in the annual number of clinical trials and drugs being tested, with a sharp increase after 2018. About the disease types of the 331 trials, Parkinson disease (young-onset, early-onset) (86, 26%), hemophilia (70, 21%), homozygote hypercholesterolemia (60, 18%) were the most common. Furthermore, it was also observed that the largest number of clinical trial units for rare disease in east China (90, 41%) and the smallest number located in northwest China (18, 6%) and northeast China (18, 6%).

**Conclusions:**

The growth trends illustrate the progress in clinical trial and drug development of rare diseases from 2013 to 2021. However, promoting orphan drugs development still is an important issue in China; at the same time, further efforts should be made for meet the unmet needs of disease types and balance the uneven distribution of medical resources for clinical trial on rare diseases.

## Introduction

There are more than 7000 rare diseases in the world, affecting more than 300 million people worldwide [1].The definition of rare diseases varies around the world. The United States defines rare diseases as those with fewer than 200,000 people per year (or less than 1/1500 of the population). The European Union defines the rare diseases as life-threatening or chronic progressive diseases with prevalence rates below 5/10000, while requiring special interventions. Japan defines rare diseases as those affecting less than 50,000 people (or 1/2500 of the population) [2, 3]. However, an official definition of rare disease in China has not been given until now. In 2018, China released the first catalogue of rare diseases, involving 121 rare diseases [4]. The catalogue is selected by authoritative experts according to certain working procedures based on the prevalence of diseases in China, level of diagnosis and treatment, burden of disease, level of health care and international experience. Notably, the list of the 121 rare diseases do not include cancers with rare mutations.

The approved list of orphan drugs is limited due to many hurdles in the processes of drug development. A study showed that a total of 133 orphan drugs covering 179 rare diseases, were approved in the EU in 2015 [5]. At the same time, in the United States, 415 orphan drugs (covering 521 rare diseases) have been approved [5]. In contrast, only a fraction of the approvals of EMA and FDA has been taken over by the Chinese regulatory authority [6]. For example, only 27 out of 60 FDA-approved orphan drugs were approved in China. Similarly, 8 out of 27 EMA-approved orphan drugs were approved in China [7].

A critical stage for drug development is to carry out clinical trials. Notably, clinical trials of rare diseases are even more difficult to implement due to multiple challenges, such as clinical trial design, patient recruitment, regulatory challenges [8, 9]. In recent years, research on the status and the trend of clinical trials for rare diseases has attracted more and more attention [10]. Through these studies, a lot of insightful analysis has been made on clinical trials of rare diseases, which provides useful reference for orphan drug development [11–13]. Most of these studies on rare diseases are based on databases such as the ClinicalTrials.gov registry and EU Clinical Trials Register. But analysis on clinical trials of rare diseases based on official databases in China are rare.

Understanding the current status of clinical trials of rare diseases is helpful to recognize and avoid the pitfalls during orphan drug development, and finally to make patients with rare diseases medically available. Therefore, this present study aims to explore the current status and trend of clinical trials of rare diseases in China through specific data analysis based on the official platform, to provide insight on improving clinical development of orphan drugs, identifying unmet clinical needs, to provide essential supportive data for policy makers and other stakeholders.

## Methods

### Data source

Clinical trials data of orphan drugs from Jan 1, 2013 to Dec 31, 2021 were extracted from the Drug Clinical Trials and Information Registration Platform (DCTIRP, http://www.chinadrugtrials.org.cn) of National Medical Products Administration (NMPA), which is an integrated platform for clinical trial registration and social publicity for drugs developed in China. The platform was initially launched on November 1st, 2012. To fulfil the scientific and moral obligations of researchers and regulatory agencies, the State Food and Drug Administration issued a notice in 2013 that all drug clinical trials being done as registration trials must be registered on the DCTIRP of NMPA, including phase I–IV drug trials and bioequivalence studies (BE), which evaluate the consistency of pharmacokinetic parameters between generic chemical drugs and reference drugs.

### Data collection

The name of 121 rare diseases in the first catalogue of rare diseases, both in Chinese and English, were used as the keywords to be searched on the platform. Diseases with multiple Chinese names were searched with all the names available, with duplicated entries removed accordingly. From January 1, 2013 to December 31, 2021, a total of 331 clinical trials of orphan drugs were extracted out from the DCTIRP of NMPA .

The clinical trial registration information extracted from the database includes, (1) basic information: registration number, initial publication date, applicant, participating agencies; (2) management information: trial status (phase I–IV trials and BE), availability of the Data Security Monitoring Committee (DMC) and trial injury insurance for subjects; (3) scientific information: tested drugs, drug types (chemical drugs and biological products), trial scope (domestic trial or international trial). Abnormal or missing data was corrected or imputed according to the original data.

### Data analysis

Microsoft Excel (version 16.47) was used for data management. SPSS (version 24) was used for statistical analysis. Graphic figures were then created with ArcMap 10.2 and Excel. Basic statistical description of count data was presented as frequency and percentage. Chi-square test was used for the comparison between groups of enumeration data, and a p value less than 0.05 was used as the threshold of statistical significance.

## Results

### Characteristics of the clinical trials data of orphan drugs

A total of 331 clinical trials of orphan drugs were extracted from the DCTIRP of NMPA from 2013 to 2021. 194 (59%) clinical trials were initiated by domestic enterprises and 137 (41%) by foreign enterprises. Nearly half of the 331 clinical trials (152, 46%) were bioequivalence studies (BE), followed by phase III trials (78, 24%), phase I trials (58, 17%), phase II trials (27, 8%), and phase IV trials (16, 5%). The proportion of BE clinical trials initiated by domestic enterprises was higher than that of foreign enterprises, and the difference was statistically significant (*p *= 0).

Domestic trials accounted for a large proportion in the total number of clinical trials of rare diseases (n = 294, 89%), compared with international trials (n = 37, 11%). The proportion of international trials initiated by foreign enterprises was higher than that of domestic enterprises (*p *= 0). Among the 331 trials, only 35 (11%) had the DMC. As shown in Table 1, 23 out 137 (17%) of the trials initiated by foreign enterprises had the data monitoring committee, which was significantly higher than that of the trials initiated by domestic enterprises (6% ) (*p *= 0). 229 trials (69%) had trial injury insurance purchased for subjects. 105 out 137 (77%) of the trials initiated by foreign enterprises had trial injury insurance for subjects, which was significantly higher than that of the trials initiated by domestic enterprises (64%) (*p *< 0.05).

**Table 1:** Characteristics of the Clinical trials data of orphan drugs

Item		Overall (n = 331)		Domestic enterprises (n = 194)		Foreign enterprises (n = 137)		χ^2^	*P* value
Study phase	Phase I	58	17%	32	16%	26	19%	47.701	0
	Phase II	27	8%	9	5%	18	13%		
	Phase III	78	24%	37	19%	41	30%		
	Phase IV	16	5%	1	1%	15	11%		
	BE	152	46%	115	59%	37	27%		
Trial scope	Domestic trial	294	89%	183	94%	111	81%	14.323	0
	International trial	37	11%	11	6%	26	19%		
DMC	Yes	35	11%	12	6%	23	17%	9.546	0
	No	296	89%	182	94%	114	83%		
Insurance	Yes	229	69%	124	64%	105	77%	6.098	0.014
	No	102	31%	70	36%	32	23%		

Two-sided *p* value from Chi-square tests for comparison of proportion.

### Time trends of initiated trials

Overall, the annual number of clinical trials of orphan drugs was on the rise from 2013 to 2021 (Mann-kedall test, *p *= 0.0025 ), with an average annual growth rate of 61%. Notably, the number of clinical trials increased significantly in 2021, with an increase of 70%, compared with the number in 2020.

**Fig. 1:**
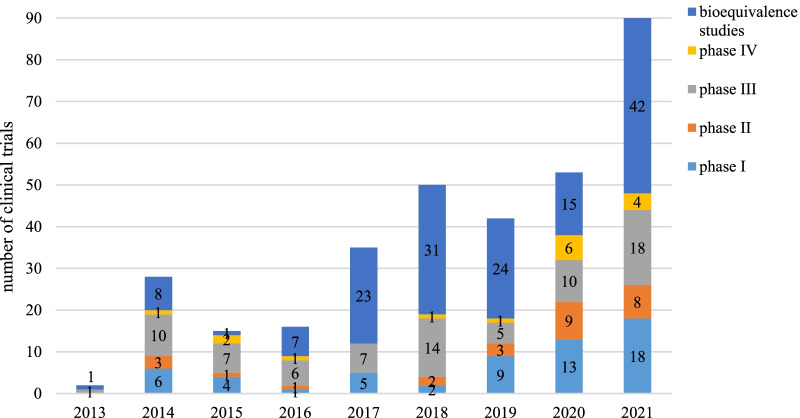
Annual numbers of clinical trials of orphan drugs by study phase

The total number of phase I and phase II clinical trials showed a steady increase from 2018 to 2021, with an annual increase of 87%. The number of BE were gradually decreased, with an average change of 31% per year from 2018 to 2020. But the number of BE rose significantly in 2021, with an increase of 180%, compared with the number in 2020 (Fig. 1).

### Time trends of tested drugs

**Fig. 2:**
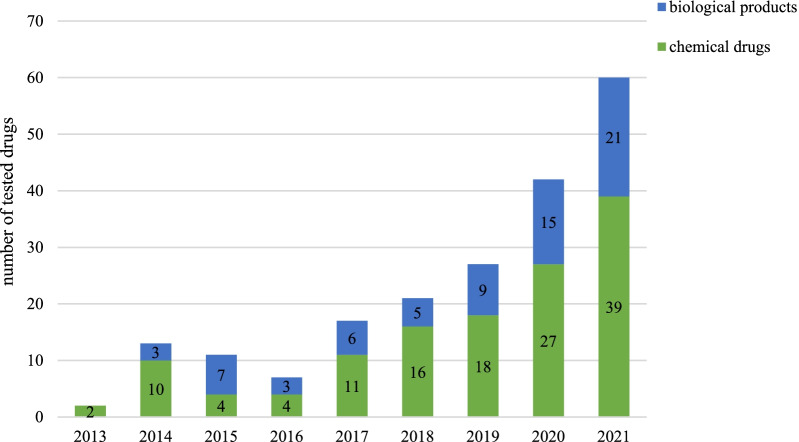
Annual numbers of tested drugs by drug type

124 drugs were tested in clinical trials from 2013 to 2021. Of the 124 drugs tested, 82 (66%) were chemical drugs, and 42 (34%) were biological products. The number of drugs tested increased annually with a significant average annual increase of 53% (*p* = 0.0025). From 2019 to 2021, the number of chemical drugs gradually increased, with an average annual increase of 47%. And the number of biological products gradually increased, with an average change of 53% per year. In addition, the numbers of chemical drugs and biological products increased in 2021, with increases of 44% and 40%, respectively, compared with these numbers in 2020 (Fig. 2).

### Rare disease types in registered clinical trials

**Fig. 3:**
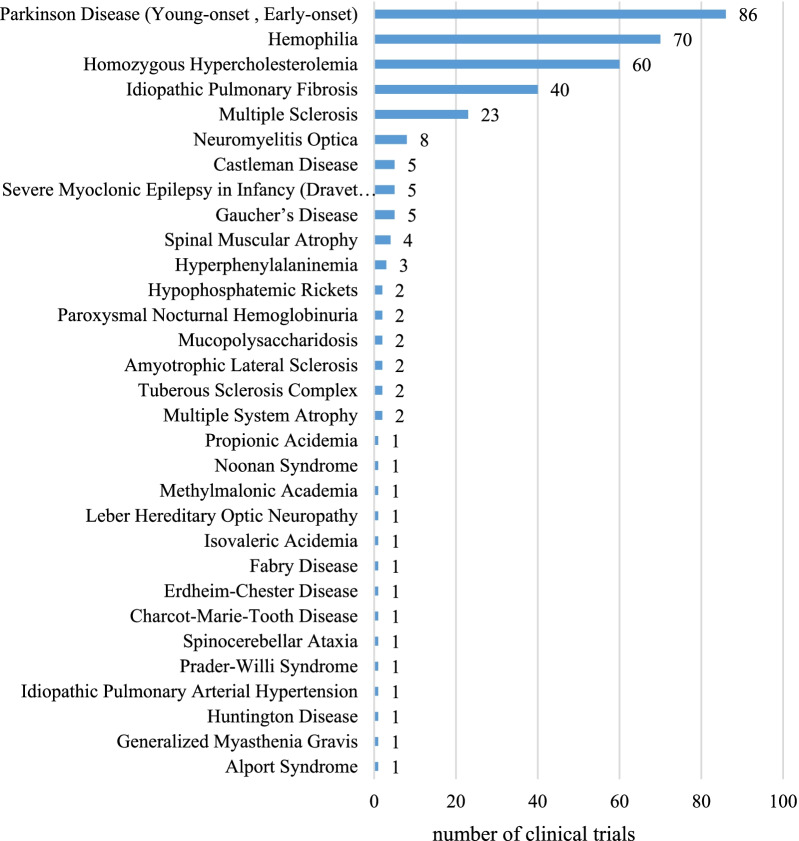
Number of clinical trials for different rare disease

331 clinical trials of orphan drugs covered 31 rare diseases in the first list. Among the 31 rare diseases, 5 had more than 20 trials each. Parkinson disease (young-onset, early-onset) was the most commonly identified rare disease with 86 (26%) trials, followed by hemophilia (70, 21%), homozygote hypercholesterolemia (60, 18%), idiopathic pulmonary fibrosis (40, 12%) and multiple sclerosis (23, 7%). The remaining 26 rare diseases had less than 10 trials each (Fig. 3).

### Geographical distribution of participating institutions

From 2013 to 2021, 281 clinical trial units were involved as the sites for clinical trials of orphan drugs in China, covering 32 provincial administrative regions. Beijing had the largest number of clinical trial units (29, 10%), followed by Shanghai (23, 8%), Jiangsu (22, 8%), and Guangdong (21, 7%). In general, the largest numbers of clinical trial units of orphan drugs were located in east China (90, 32%), followed by north China (52, 19%) and south China (48, 17%). The smallest numbers of clinical trial units were located in northwest China (18, 6%) and northeast China (18, 6%).

**Fig. 4:**
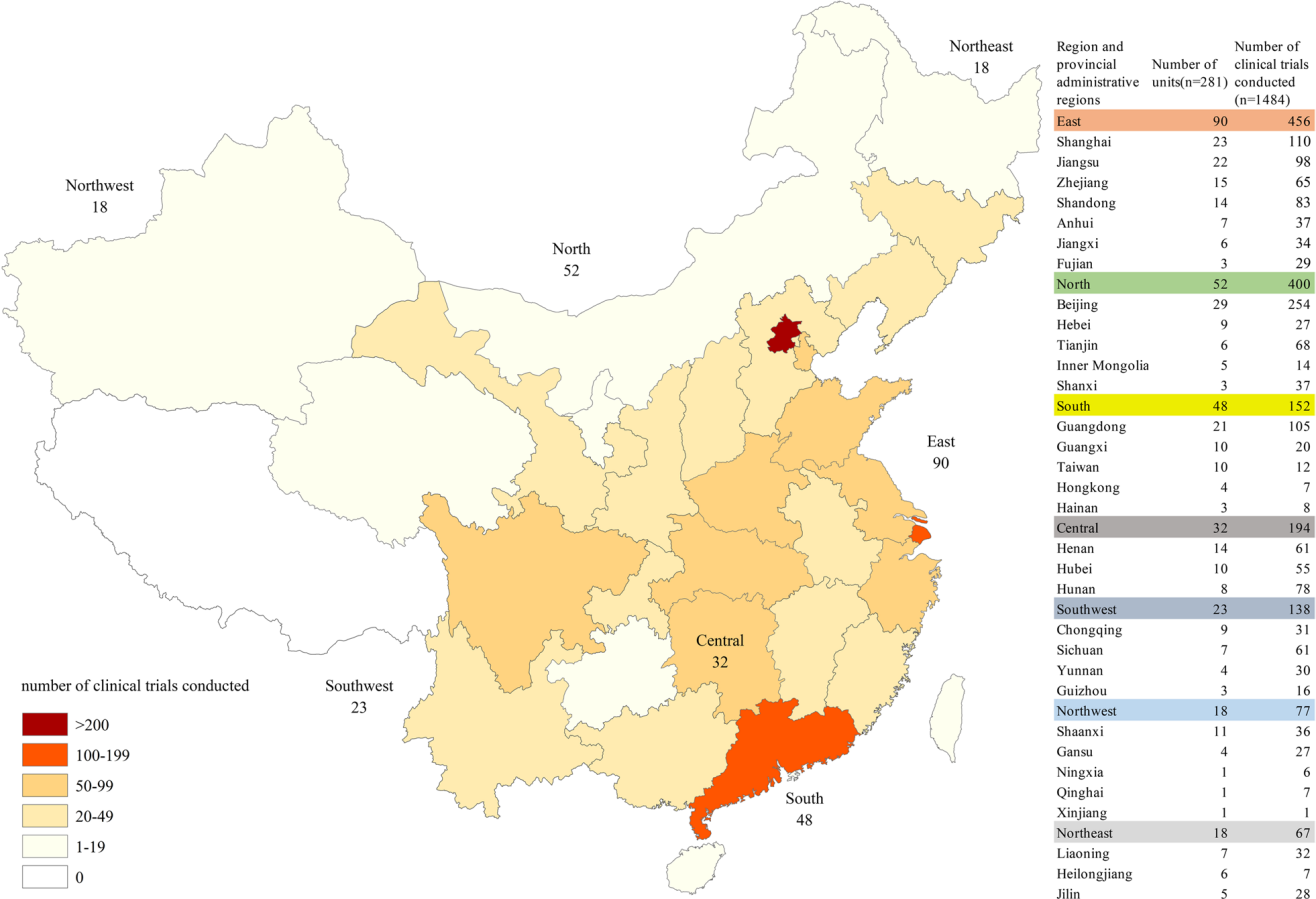
Geographical distribution of clinical trial units of clinical trials of rare disease

In terms of the number of clinical trials conducted, units in Beijing had the largest number (254, 17%), followed by units in Shanghai (110, 7% ) and Guangdong (105, 7%). Consistently, the largest numbers of clinical trials conducted by units were also located in east China (456, 31%), followed by north China (400, 27%). The smallest numbers of clinical trials conducted by units were located in northwest (77, 5%) and northeast China (67, 5%) (Fig. 4).

## Discussion

This is a comprehensive article on the status of rare disease clinical trials in China. The time trends of initiated clinical trials and tested drugs indicate that certain progress has been made in the clinical development of orphan drugs in China from 2013 to 2021. Notably, the limited number of clinical trials for most rare diseases, and the uneven geographical distribution of clinical trial units may provide potential improvement goals for policy makers and companies involved in drug development for rare diseases.

Bioequivalence studies accounted for a great portion, mostly initiated by domestic enterprises. Many pharmaceutical companies developed generic versions of the same drug, especially for Parkinson' s disease and homozygous familial hypercholesterolemia. On the one hand, the successful development of generic drugs can solve the unmet clinical needs of patients with rare diseases in China and reduce their economic burden. On the other hand, the phenomenon is also a reflection of the lack of innovation of some Chinese pharmaceutical companies. Many pharmaceutical companies tend to develop generic drugs, instead of original drugs, mainly due to the following reasons. First of all, the lack of epidemiological data for some rare diseases has led to unknown market capacity and increased risks of research and development [14]. In addition, the basic research on rare diseases in China is weak, which increases the difficulty of preclinical research. Besides, the development of generic drugs for some rare diseases urgently needed in clinic is also encouraged by the government. In October 2019, the National Health Commission issued the first batch of 33 “National Encouraging Catalogs of Imitation Drugs”, including 6 orphan drugs for the treatment of 4 rare diseases [15].

The coverage of DMC and insurance of clinical trials initiated by domestic enterprises is lower than that of foreign enterprises. DMC is an independent committee composed of experts with relevant knowledge and experience, which reviews the process and data of clinical trials, aiming to ensure the safety of subjects and the rationality and scientificity of continuing trials [16] . A study showed that, the appointment of DMC was more common in rare disease trials as compared other clinical trials (53% vs. 41%) [17]. In addition, the insurance coverage rate of rare disease trials (69%) is higher than that of general clinical trials (38.2%). These differences may be due to more clinical trials of generic drugs initiated by domestic enterprises, which rarely require DMC and insurance. Furthermore, these may be partially due to the incomplete regulatory system in China that does not mandate DMCs or insurances for clinical trials. In order to strengthen the protection of subjects, it is suggested to further improve the laws and regulations related to clinical trial.

The gradual growth of the annual number of clinical trials for rare diseases suggested the contribution of Chinese pharmaceutical industry to the global pipeline from 2013 to 2021. According to the data released by the DCTIRP, the average annual growth rate of the total number of clinical trials for all diseases was lower than that of the number of clinical trials for rare diseases from 2013 to 2021 (33% vs. 61%). The number of phase I and II clinical trials has increased significantly, consistently with an increase of orphan drugs at preliminary clinical stage. In order to mobilize pharmaceutical companies to develop orphan drugs, the Chinese government has issued many supportive policies [18, 19]. In particular, in 2015, a milestone policy was issued on promoting drug innovation for rare diseases and the efficiency of clinical trials [20]. Moreover, in order to ensure generic drug safety and efficacy, the China Food and Drug Administration required the quality and efficacy consistency evaluation of generic drugs, including generic drugs for rare diseases since 2016 [21]. In addition, in order to promote the clinical research and development of rare disease drugs, in January 2022, CDE released the “Technical Guidelines for Clinical Development of Rare Disease Drugs” [22]. Clinical development of drugs for rare diseases is more difficult than for common diseases [8, 9]. This guideline encourages more flexible designs and the best use of limited patient data and provides recommendations for the selection of clinical/surrogate endpoints.

Increases were seen in the number of drugs being tested, both chemical drugs and biological products. According to the NMPA website, 41 (33%) of the 124 drugs for rare diseases in the study have been marketed in China as of December 30, 2021. Several policies have been promulgated to improve the availability of orphan drugs in China. In terms of drug registration, a green channel has been established for orphan drugs, with priority review and approval in 2019 [23]. In 2020, the NMPA required a 70-day review time limit for orphan drugs that have been marketed overseas but have not been marketed in China and are urgently needed clinically [24]. For some ultra-rare diseases, a more flexible strategy should be adopted. For example, the U.S. Food and Drug Administration (FDA) approved uridine triacetate for the treatment of HOA (hereditary orotic aciduria) based on a 6-week clinical trial based on 4 patients [25].

The number of clinical trials varies widely among different rare diseases. Data on incidence/prevalence of rare diseases in China are limited [14]. Thus, only a few data can be used to explore the comparison between the epidemiology of rare diseases and the number of clinical trials in China. Up to April 5, 2022, the number of rare patients registered on the National Rare Diseases Registry System of China (NRDRS) has reached 68684. The top 5 of diseases registered on the NRDRS are hemophilia, Duchenne/Becker muscular dystrophy, spinocerebellar ataxia, rare pulmonary hypertension, autosomal dominant polycystic kidney disease. 70 clinical trials were for hemophilia, but only 1 each for spinocerebellar ataxia and rare pulmonary hypertension. In addition, 98 out of the 121 rare diseases need drug treatment [26]. The clinical trials for the remaining 67 rare diseases have not been carried out in China, indicating the unmet needs for these patients. Notably, this situation also occurs in other countries. For example, it has been shown that the EU does not have orphan products for certain rare diseases [5]. To encourage drug development for rare diseases that do not have effective treatments yet globally is a common direction for pharmaceutical companies and governments in various countries.

Most of the clinical trial units for rare diseases are tertiary hospitals with strong research background. Peking Union Medical College Hospital has the highest number of clinical trials. In 2019, Peking Union Medical College Hospital lead in the establishment of a rare disease diagnosis and treatment cooperation network in China. In addition, Peking Union Medical College hospital is also responsible for building the NRDRS. In general, clinical trial units for rare diseases in China are mainly distributed in the developed regions, such as the northern and eastern China. However, this also reflects some problems. The distribution of medical resources for clinical research in China is quite uneven, which is partially due to the leading role of large clinical trial units required by the government [27]. In the future, the government should also consider the problem of regional imbalance, so that more institutions and greater areas can be involved in clinical trials for orphan drugs.

Based on the DCTIRP of NMPA, our systematic review elucidated the status and trend of clinical trials for rare diseases in China from 2013 to 2021. In the future, more attention should be paid to the unmet needs of some rare diseases and the drug development for these diseases. Besides, it is hoped that more institutions and greater areas can be involved in clinical trials for orphan drugs and facilitate the drug development of rare diseases.

### Limitation

The limitation of the current study is listed below. Firstly, this database is input by enterprises themselves, and there is the possibility of missing or mis-recording of data. In addition, part of the input is not standardized, which can possibly result in potential data deviation. Secondly, this study collected orphan drug clinical trials data solely registered on the DCTIRP, and did not include clinical trials initiated by investigators. Thirdly, only 121 rare diseases were retrieved, which did not take into account of clinical trials for other rare diseases. Finally, international comparisons were not completed since no equivalent reports have been found.

## Conclusion

From 2013 to 2021, the gradual growth of the annual numbers of clinical trials and drugs tested suggest the contribution of Chinese pharmaceutical industry to the global pipeline. However, promoting medical innovation still is an important issue worthy of exploration by policy makers and pharmaceutical enterprises in China; at the same time, further efforts should be made for meet the unmet needs and balance the uneven distribution of medical resources for clinical trial on rare diseases.

## Data Availability

The datasets generated and analyzed during the current study are not publicly available as they contain confidential commercial information, Limited information may be available from the corresponding author upon request but will be reviewed to protect proprietary information.
